# Retraction Note: Non-invasive vagus nerve stimulation improves clinical and molecular biomarkers of Parkinson’s disease in patients with freezing of gait

**DOI:** 10.1038/s41531-022-00424-6

**Published:** 2022-11-03

**Authors:** Banashree Mondal, Supriyo Choudhury, Rebecca Banerjee, Akash Roy, Koustav Chatterjee, Purba Basu, Ravi Singh, Saptak Halder, Shantanu Shubham, Stuart N. Baker, Mark R. Baker, Hrishikesh Kumar

**Affiliations:** 1 Institute of Neurosciences Kolkata, Kolkata, India; 2 Faculty of Medical Sciences, Newcastle University, Newcastle upon Tyne, UK; 3 Department of Neurology, Royal Victoria Infirmary, Newcastle upon Tyne, UK; 4 Department of Clinical Neurophysiology, Royal Victoria Infirmary, Newcastle, UK

**Keywords:** Parkinson's disease, Predictive markers

Retraction to: *npj Parkinson’s Disease* 10.1038/s41531-021-00190-x, published online 27 May 2021

The Editor in Chief has retracted this article. After publication, concerns were raised regarding irregularities in the data in Table 2 and Fig 4. After re-examining the data, the authors provided a correction. However, independent editorial assessment of this correction showed the original conclusions presented in the article are not adequately supported. None of the authors agree with this retraction.

